# Non-canonical functions of spliceosome components in cancer progression

**DOI:** 10.1038/s41419-022-05470-9

**Published:** 2023-02-02

**Authors:** Olga M. Ivanova, Ksenia S. Anufrieva, Anastasia N. Kazakova, Irina K. Malyants, Polina V. Shnaider, Maria M. Lukina, Victoria O. Shender

**Affiliations:** 1 Center for Precision Genome Editing and Genetic Technologies for Biomedicine, Federal Research and Clinical Center of Physical-Chemical Medicine of Federal Medical Biological Agency, Moscow, 119435 Russian Federation; 2 Federal Research and Clinical Center of Physical-Chemical Medicine of the Federal Medical and Biological Agency, Moscow, 119435 Russian Federation; 3 Institute for Regenerative Medicine, Sechenov University, Moscow, 119991 Russian Federation; 4 Moscow Institute of Physics and Technology (State University), Dolgoprudny, 141701 Russian Federation; 5 Faculty of Chemical-Pharmaceutical Technologies and Biomedical Drugs, Mendeleev University of Chemical Technology of Russia, Moscow, 125047 Russian Federation; 6 Faculty of Biology, Lomonosov Moscow State University, Moscow, 119991 Russian Federation; 7 Shemyakin-Ovchinnikov Institute of Bioorganic Chemistry of the Russian Academy of Sciences, Moscow, 117997 Russian Federation

**Keywords:** Cancer, Mechanisms of disease

## Abstract

Dysregulation of pre-mRNA splicing is a common hallmark of cancer cells and it is associated with altered expression, localization, and mutations of the components of the splicing machinery. In the last few years, it has been elucidated that spliceosome components can also influence cellular processes in a splicing-independent manner. Here, we analyze open source data to understand the effect of the knockdown of splicing factors in human cells on the expression and splicing of genes relevant to cell proliferation, migration, cell cycle regulation, DNA repair, and cell death. We supplement this information with a comprehensive literature review of non-canonical functions of splicing factors linked to cancer progression. We also specifically discuss the involvement of splicing factors in intercellular communication and known autoregulatory mechanisms in restoring their levels in cells. Finally, we discuss strategies to target components of the spliceosome machinery that are promising for anticancer therapy. Altogether, this review greatly expands understanding of the role of spliceosome proteins in cancer progression.

## Facts

- To overcome various stresses, cancer cells may exploit not only splicing activity of spliceosome components but also their splicing-independent functions.
- Spliceosome components are involved in intercellular communication.
- Splicing-independent functions of spliceosome components also need to be taken into account to counteract their oncogenic activity.

## Open questions

- Do all spliceosome components have direct functions in other cellular processes besides the pre-mRNA splicing? What are the exact mechanisms?
- How do cancer cells balance canonical and non-canonical functions of spliceosome components to regulate stress response?
- Being agents of intercellular communication how do spliceosome components change processes inside cells in the tumor microenvironment?
- Is it possible to improve the efficiency of cancer therapies by targeting spliceosome components?

## Introduction

**Fig. 1:**
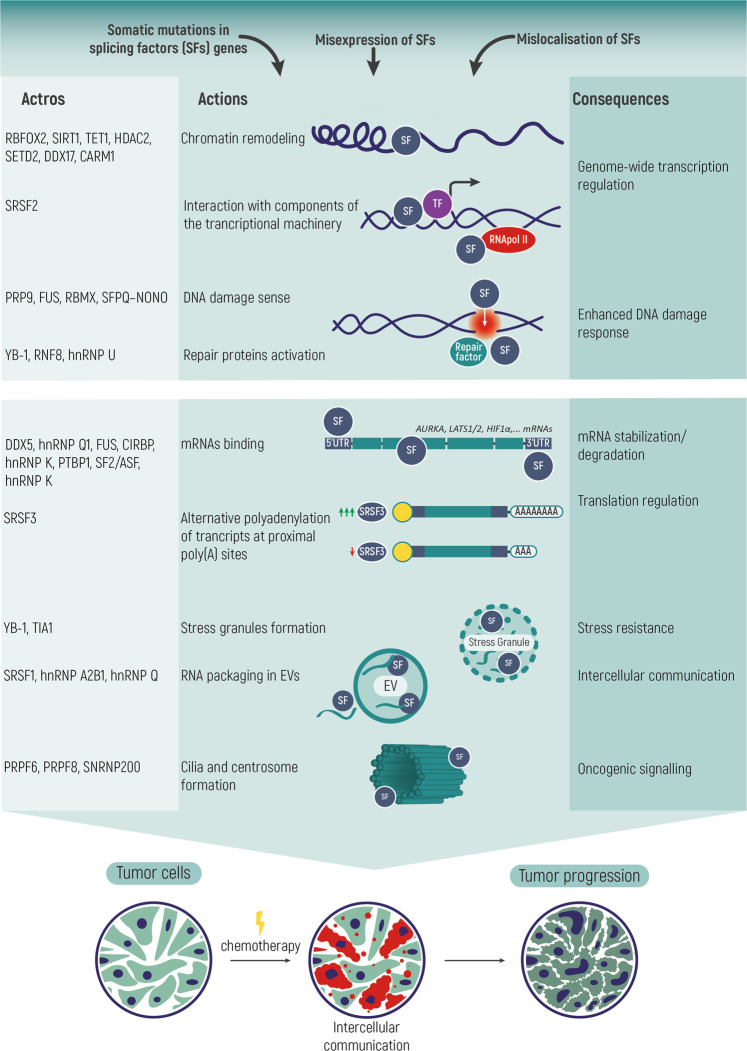
Non-canonical functions of spliceosome components in cancer progression. Splicing perturbations are common in cancer and are associated with mutations, altered expression and/or localization of the components of the splicing machinery. Moreover, spliceosome components can be secreted by dying tumor cells as part of extracellular vesicles and penetrate recipient cells, thus ensuring their greater resistance to ongoing therapy. Both splicing factors and small nuclear RNAs may have functions not only related to pre-mRNA splicing, which contribute to every hallmark of cancer and all kinds of cellular processes. SF splicing factors, TF transcription factors, UTR untranslated region, EV extracellular vesicles.

Multiple datasets indicate that somatic mutations, aberrant expression, and/or localization of spliceosome components in cancer cells lead to various defects in pre-mRNA splicing and other cellular functions, which can contribute to enhanced tumor cell proliferation, invasion, metastasis, chemoresistance, and inhibition of apoptosis [1–3]. Various studies and reviews have focused on the analyses of somatic mutations in the splicing machinery in malignant tumors compared with corresponding normal tissues, or discussed splice isoforms of specific genes that may have antagonistic functions essential for cancer progression [4, 5]. However, several recent thought-provoking studies have suggested that spliceosome components (both splicing factors and small nuclear RNAs) may have functions beyond pre-mRNA splicing. Recently it has been shown that spliceosome components can be secreted by dying tumor cells as part of extracellular vesicles and penetrate recipient cells, thus ensuring their greater resistance to ongoing therapy [6–8]. Since the functions and activity of splicing factors depend on their abundance and localization in a cell, the expression of splicing factors is tightly regulated, including autoregulation mechanisms. However, in many cases, the impact of such autoregulatory loops on splicing dynamics remains unclear. For a deeper view, we have gathered here the known examples. Perturbations in alternative splicing in cancer cells are of high interest in terms of anticancer therapy. But there is still a lack of understanding of tumor-associated splicing regulation despite numerous studies. In this review, we discuss how disturbances in the expression, abundance, and localization of various components of the splicing machinery affect cellular processes (proliferation, cell cycle, cell division, cell death, DNA repair, etc.) important for cancer progression (Fig. 1).

## Splicing machinery

The processes of constitutive and alternative splicing (AS) are catalyzed by the spliceosome, a dynamic macromolecular complex which includes up to five unique small nuclear RNAs (snRNAs) and >200 different protein factors depending on stage of spliceosome assembly [9]. snRNAs are a class of short (about 150 nt on average) non-coding RNAs highly abundant in the cell. They mainly localize in the nucleus and perform functions associated with pre-mRNA splicing and processing. Each snRNA is associated with seven spliceosome core proteins (Sm proteins; or Lsm proteins in the case of U6 snRNA) and a number of other specific, highly conserved proteins (e.g., U1-70K, U2 snRNP A’, NHP2L1/SNU13, U5-40K), thus forming small nuclear ribonucleoproteins (snRNPs) U1, U2, U4/U6, and U5 [10, 11]. Additional proteins also copurify with the core components of splicing machinery throughout its assembly and catalytic stages. These include (1) various regulatory proteins, such as Serine/Arginine-rich (SR) proteins and heterogeneous nuclear ribonucleoproteins (hnRNPs) which interact with splicing enhancers and silencers, respectively, and have broadly antagonistic effects on alternative splicing, and (2) proteins with enzymatic functions that may act as conformational switches, such as GTPase EFTUD2, methyltransferase PRMT5, and several RNA helicases including proteins with DExD/H-box [10]. Also many spliceosome proteins are pre-organized into large functional ensembles, for example, the retention and splicing (RES) complex, the splicing essential PRP19/CDC5L complex, and the pentameric intron-binding complex (IBC) [12].

In addition to the main spliceosome, a less abundant minor spliceosome also functions in the cell. This minor spliceosome is composed of snRNPs U11, U12, U4atac, U6atac, and U5 and is responsible for splicing U12-type introns [13], which makes up about 0.5–1% of all introns in the human genome. Many genes with such introns encode proteins involved in DNA replication and repair, RNA processing and translation, cytoskeletal organization, and vesicular transport [14].

Finally, it should be noted that many spliceosome proteins have redundant functions and/or are weakly associated with spliceosome, indicating that each of them is not required to splice every pre-mRNA substrate. Moreover, depending on the context, the same protein can both inhibit or activate splicing, which provides fine regulation of this process [15, 16]. Besides canonical splicing, splicing of microexons, recursive splicing and biogenesis of circular and chimeric RNAs through back-splicing and trans-splicing processes are also taken place in cells often involving the same molecular players [17]. Such a diversity of splicing machinery makes it highly flexible to accurately regulate splicing outcomes in a cell type- and intron-specific manner [18].

## Non-canonical functions of spliceosome components

In addition to pre-mRNA splicing, spliceosome components are involved in other cellular processes (Fig. 1). snRNAs play an important role in multiple aspects of RNA metabolism: mRNA transcription, stabilization and degradation [19], regulation of gene expression [20], 3'-end processing of non-polyadenylated mRNA of histones [21], and recruitment of long non-coding RNAs to chromatin [22]. New data shows evidence that splicing factors also have a range of additional functions that are not associated with the splicing process. Many splicing factors have been demonstrated to be able to directly bind chromatin in promoter regions of the genes [23, 24]. For example, the RBFOX2 protein associated with nascent RNA recruits to chromatin the chromatin remodeling Polycomb complex 2 proteins and thus mediates genome-wide transcriptional regulation in mammalian cells [25]. SR proteins, such as SRSF2, have been shown to interact with components of the transcriptional machinery to mediate transcription activation [26, 27]. In general, new observations support a model in which specific splicing factors recruit core transcription machinery in close proximity to transcripts when they are being transcribed, increasing RNA polymerase II occupancy and activity of nearby promoters [28]. Some splicing factors are involved in DNA repair and replication of the telomeric regions, as well as maintenance of the genome stability through regulation of appearance and resolution of R-loops formed between nascent transcripts and complementary DNA strand during transcription [29–32]. Spliceosome proteins combine the functions of genome stability regulators and splicing participants, which is made possible by their interaction with both chromatin [23, 33] and transcribed mRNAs. Seemingly, this allows for efficient coordination and rapid switching between the functions depending on the immediate needs of the cell. Besides, splicing factors have been shown to be actively involved in regulation of the M phase of the cell cycle independently of their main functions [34]. Also, spliceosome proteins can regulate gene expression through control of not only mRNA processing but also the export of transcripts from the nucleus to the cytoplasm [35–38]. Splicing factors are actively involved in various aspects of RNA molecule biogenesis performing microRNA processing [39], regulating recognition of polyadenylation sites [40], mRNA stabilization [41–43], and degradation [44]. In addition to direct or indirect interaction of splicing factors with DNA and RNA in the cell nucleus, splicing factors are also involved in the formation of stress granules in the cytoplasm (YB-1 [45], TIA1 [46]) or involved in packing of various RNA classes in extracellular vesicles, like SRSF1 [47], hnRNP A2B1 [48], hnRNP Q [49, 50]. A number of spliceosome proteins have also been shown to play a role in organization and functioning of cilia and centrosomes. For example, PRPF6, PRPF8, and SNRNP200 proteins of U5 snRNP are localized in the ciliary basal body or the centrosome in cytoplasm [51]. Also, nucleo-cytoplasmic shuttling of the SRSF1 splicing factor is critical for active ciliogenesis, as the lack of cytoplasmic SRSF1 is known to repress cilia-related mRNA transcripts [52].

Thus, many proteins involved in pre-mRNA splicing are multifunctional proteins and many of them can also be determined as moonlighting proteins as they also participate in many other cellular processes, such as DNA repair, transcription and translation regulation, cell cycle progression, and cell senescence. Next, we will analyze in more detail the role of spliceosome components in these processes.

## Alterations in spliceosome components levels affect cellular processes associated with cancer progression

Altered levels of spliceosome components can significantly contribute to the acquisition of chemoresistance and more aggressive phenotype of cancer cells [3, 53]. Typical effects include increased tumor cell proliferation, angiogenesis, invasion, metastases, metabolism, and inhibition of apoptosis. Examples of molecular mechanisms of cancer progression associated with impairment of alternative splicing recently have been well reviewed by Du and co-authors [54]. In this review, we focused on functions of spliceosome proteins not associated with alternative splicing of mediator genes (Table 1).

**Table 1:** Impact of splicing factors overexpression/mislocalization on cellular processes in cancer cells.

Cellular process	Protein	Role in mRNA splicing (UniProt information)	Mechanism mediated by non-canonical functions	Pro-oncogenic effect upon	Ref.
Cell proliferation, migration and invasion	**FUS**	Binds both single-stranded and double-stranded DNA and promotes ATP-independent annealing of complementary single-stranded DNAs and D-loop formation in superhelical double-stranded DNA. May play a role in maintenance of genomic integrity.	Binds to *LATS1/2* mRNA, which ensures its stabilization and activation of the Hippo signaling pathway.	Downexpression	[67]
	**hnRNP Q1**	Heterogenous nuclear ribonucleoprotein implicated in mRNA processing mechanisms.	Binds to 5'-untranslated regions of *Aurora-A* mRNA thus regulating its translation, which enhances cell proliferation.	Overexpression	[65, 79]
	**RBFOX2**	RNA-binding protein that regulates alternative splicing events by binding to 5'-UGCAUGU-3' elements. Prevents binding of U2AF2 to the 3'-splice site.	Recruits Polycomb complex 2 proteins, which lead to chromatin remodeling and genome-wide transcription regulation.	Ambiguously	[25]
	**SRSF2**	Necessary for the splicing of pre-mRNA. It is required for formation of the earliest ATP-dependent splicing complex and interacts with spliceosome components bound to both the 5'- and 3'-splice sites during spliceosome assembly. It also is required for ATP-dependent interactions of both U1 and U2 snRNPs with pre-mRNA. Interacts with other spliceosome components, via the RS domains, to form a bridge between the 5'- and 3'-splice site binding components, U1 snRNP and U2AF.	Interacts with components of the transcriptional machinery thus regulating transcription.	Ambiguously	[26, 27]
Cell cycle progression, mitosis	**DDX5**	Involved in the alternative regulation of pre-mRNA splicing; its RNA helicase activity is necessary for increasing tau exon 10 inclusion and occurs in a RBM4-dependent manner. Binds to the tau pre-mRNA in the stem-loop region downstream of exon 10.	Binds to a noncoding RNA *SUNO1*, stabilizes RNA polymerase II on chromatin and enhances transcription of cell cycle genes; Binds with cyclin genes mRNAs, regulating their stability and nuclear export.	Overexpression	[78]
	**RBM10**	May be involved in post-transcriptional processing, most probably in mRNA splicing. Binds to RNA homopolymers, with a preference for poly(G) and poly(U) and little for poly(A).	Regulates centriole duplication; overexpression in tumor cells leads to cell cycle arrest in the M phase and the formation of a monopolar spindle due to disturbances in centriole duplication.	Downexpression	[80]
	**SF3A2**	Subunit of the splicing factor SF3A required for ‘A’ complex assembly formed by the stable binding of U2 snRNP to the branchpoint sequence in pre-mRNA. Sequence independent binding of SF3A/SF3B complex upstream of the branch site is essential, it may anchor U2 snRNP to the pre-mRNA. May also be involved in the assembly of the ‘E’ complex.	Regulate the interaction between kinetochores, spindle microtubules, and the essential kinetochore complex Ndc80	Ambiguously	[81]
	**PRP31**	Involved in pre-mRNA splicing as component of the spliceosome. Required for the assembly of the U4/U5/U6 tri-snRNP complex, one of the building blocks of the spliceosome.			
	**EFTUD2**	Required for pre-mRNA splicing as component of the spliceosome, including pre-catalytic, catalytic and post-catalytic spliceosomal complexes	Interact with cohesin for mitotic progression.	Ambiguously	[86]
	**SNRNP200**				
DNA repair	**PRP19**	Core component of the PRP19C/Prp19 complex/NTC/Nineteen complex which is part of the spliceosome and participates in its assembly, its remodeling and is required for its activity. During assembly of the spliceosome, mediates ‘Lys-63’-linked polyubiquitination of the U4 spliceosomal protein PRPF3. Ubiquitination of PRPF3 allows its recognition by the U5 component PRPF8 and stabilizes the U4/U5/U6 tri-snRNP spliceosomal complex.	Ubiquitinates RPA, acts as sensor for SSBs; enhances DNA damage response. Requied for sister chromatid cohesion.	Downexpression	[31]
	**FUS**	see above	Interacts with FDAC1, acts as sensor for DSBs; enhances DNA damage response.	Downexpression	[100]
	**RBMX/hnRNP G**	RNA-binding protein that plays several role in the regulation of pre- and post-transcriptional processes. Implicated in tissue-specific regulation of gene transcription and alternative splicing of several pre-mRNAs. Associates with nascent mRNAs transcribed by RNA polymerase II. Component of the supraspliceosome complex that regulates pre-mRNA alternative splice site selection. Can either activate or suppress exon inclusion.	Stabilizes paired DNA ends; enhances DNA damage response.	Downexpression	[108]
	**SFPQ–NONO**		Stabilizes paired DNA ends; enhances DNA damage response.	Downexpression	[107]
	**YB-1**	Mediates pre-mRNA alternative splicing regulation. Binds to splice sites in pre-mRNA and regulates splice site selection.	Participates in almost all types of DNA repair; enhances DNA damage response.	Downexpression	[102]
	**RNF8**	E3 ubiquitin-protein ligase.	Ubiquitinates DNA in damage lesions, recruits repair factors; enhances DNA damage response.	Downexpression	[104]
	**hnRNP U**	Binds to pre-mRNA, is required for normal pre-mRNA splicing of many targets.	Stimulates activity of DNA glycosylase NEIL1; enhances DNA damage response.	Downexpression	[105]
	**RNF113A**	Required for pre-mRNA splicing as component of the spliceosome. E3 ubiquitin-protein ligase that catalyzes the transfer of ubiquitin onto target proteins.	Stabilizes the levels of an antiapoptotic protein MCL-1 and prevents cell death.	Overexpression	[93]
	**CIRBP**	Cold-inducible mRNA binding protein that plays a protective role in the genotoxic stress response by stabilizing transcripts of genes involved in cell survival.	Induces expression of HIF-1α via binding to the 3'-UTR of its mRNA to increase the mRNA stability.	Ambiguously	[94]
Cell death	**USP39**	May play a role in mRNA splicing.	Deubiquitinates and stabilizes CHK2; regulates apoptosis.	Ambiguously	[111, 112]
	**DHX32**	DEAD box protein, putative RNA helicase. It is implicated in a number of cellular processes involving alteration of RNA secondary structure such as translation initiation, nuclear and mitochondrial splicing, and ribosome and spliceosome assembly.	mRNA processing of mitochondrial RNAs, impacts on mitochondria-mediated apoptosis.	Downexpression	[110]
	**hnRNP K**	One of the major pre-mRNA-binding proteins. Binds tenaciously to poly(C) sequences. Likely to play a role in the nuclear metabolism of hnRNAs, particularly for pre-mRNAs that contain cytidine-rich sequences.	Binds to the CU-rich region in thymidine phosphorylase mRNA, thus stabilizing they, and increasing protein levels of thymidine phosphorylase, which help to resist hypoxia-induced apoptosis.	Ambiguously	[113]
	**SRSF1**	Plays a role in preventing exon skipping, ensuring the accuracy of splicing and regulating alternative splicing. Interacts with other spliceosome components, via the RS domains, to form a bridge between the 5'- and 3'-splice site binding components, U1 snRNP and U2AF. Can stimulate binding of U1 snRNP to a 5'-splice site-containing pre-mRNA.	Stabilizes mRNA of the anti-apoptotic protein survivin, increasing its translation.	Overexpression	[43]
	**SIRT1**	NAD-dependent protein deacetylase.	Regulates processes such as apoptosis by deacetylating key proteins.	Ambiguously	[115, 116]
	**DDX5**	see above	Binds to autophagic receptor p62, promoting its activation; decreases p62/TRAF6-mediated lysine 63-linked ubiquitination of mammalian target of rapamycin (mTOR); and subsequently inhibits the mTOR signaling pathway.	Overexpression	[117]
Cellular senescence	**DDX24**	ATP-dependent RNA helicase.	Interacts with p300, increases p53 acetylation, induces cell cycle arrest and cellular senescence.	Ambiguously	[128]
	**SRSF3**	May be involved in RNA processing in relation with cellular proliferation and/or maturation.	Alternative polyadenylation of transcripts at proximal poly(A) sites; enhances production of senescence-associated proteins.	Ambiguously	[127]

**Fig. 2:**
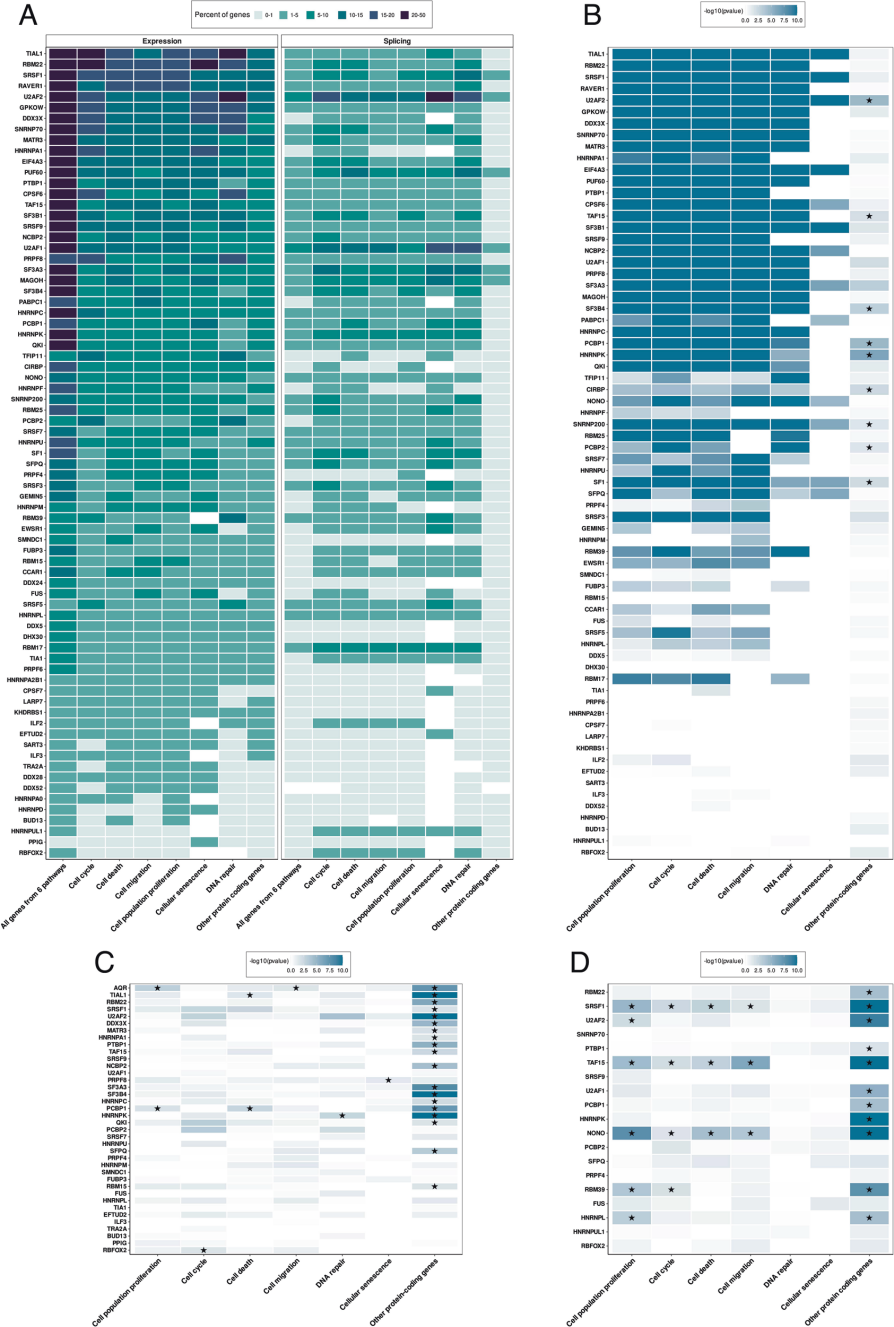
Impact of splicing factors knockdown on gene expression and pre-mRNA splicing in the HepG2 cell line. **A** The heat map shows the percent of genes associated with indicated Gene Ontology terms that have changed their expression or splicing in response to splicing factors knockdown in HepG2 cells (percent is indicated by the gradient green color). **B** The overlap between genes affected by alternative splicing and differentially expressed genes upon knockdown for each splicing factor (*p*-values are indicated by the gradient blue color). **C** The overlap between genes with significantly enriched eCLIP signal and genes with significantly altered expression upon knockdown for each splicing factor (*p*-values are indicated by the gradient blue color). **D** The overlap between genes with significantly enriched ChIP-seq signal in promoter regions and differentially expressed genes upon knockdown for each splicing factor (*p*-values are indicated by the gradient blue color). All data were obtained via the analysis of the publicly available datasets from the ENCODE project. Significance was determined by two-sided Fisher’s exact test (*p*-value < 0.05 and the odd ratio confidence interval should not include 1). The significant enrichment is indicated by an asterisk. “All genes from 6 pathways” include all genes from 6 Gene Ontology (GO) terms: “Cell population proliferation”, “Cell migration”, “DNA repair”, “Cell senescence”, “Cell cycle”. “Other protein-coding genes” means all protein-coding genes not belonging to the above-mentioned GO terms. Detailed information about bioinformatics analysis of these data is provided in the Supplemental Methods.

We used publicly available datasets from the ENCODE project and analyzed how knockdown of 75 different splicing factors in liver hepatocellular carcinoma cell line HepG2 affects the expression and splicing of genes associated with cell proliferation, migration, cell cycle regulation, DNA repair, and cell death (Fig. 2A, Supplemental Methods, Supplemental Table 1). According to our analysis, the percent of genes that have been differentially expressed or spliced upon the knockdown of almost each splicing factor in HepG2 cells was higher in case of these 6 cancer-related pathways than for other protein-coding genes (Fig. 2A). Knockdown of only 12 splicing factors (HNRNPK, HNRNPL, HNRNPUL1, MAGOH, RBFOX2, RBM17, PPIG, SF3A3, SFPQ, SRSF3, U2AF1, U2AF2) leads to considerable changes in pre-mRNA splicing of genes involved in these pathways (Supplemental Table 1). Proteins SF3A3, U2AF1 and U2AF2 are directly involved in the recognition and stabilization of the branch point, therefore changes in their abundance in cells could considerably affect pre-mRNA splicing. Remarkably, we noticed that differentially expressed genes and genes affected by alternative splicing overlap weakly (Fig. 2B). This could be just another consequence of the multifunctional nature of the splicing factors.

Next, using eCLIP data from the Encode project we analyzed whether considered splicing factors bind to those transcripts whose expression but not splicing changed in response to these splicing factors’ knockdown in HepG2 cells. Interestingly, we showed the low significance of the overlap between such differentially expressed genes and mRNA targets of the same splicing factors according to RNA-seq and eCLIP-seq data, respectively (Fig. 2C, Supplemental Table 2). Considering the deep regulatory network of splicing factors, we propose that the knockdown of one splicing factor may drive changes in the expression of many other splicing factors thus affecting splicing of a broad range of mRNAs. Knockdown of splicing factors HNRNPK, PCBP1 showed a significant correlation between eCLIP and increased expression of their mRNA targets. It may be evidence of restoring of productive splicing or it can be assumed that these splicing factors may also act as RNA decay factors. Knockdown of other splicing factors, PRPF8, TIAL1, AQR showed correlation between eCLIP and decreased expression of their targeted mRNA. These splicing factors may be associated with positive regulation of the stability of mRNA targets (Fig. 2C, Supplemental Table 2).

Since it is known that splicing factors can play a direct role in promoting transcriptional elongation, we also analyzed ChIP-seq data for 19 splicing factors from the Encode project. The knockdown of 6 splicing factors (U2AF2, TAF15, SRSF1, RBM39, NONO, HNRNPL) greatly affects the expression of genes enriched in ChIP-seq analysis for the same splicing factors (Fig. 2D, Supplemental Table 3). All of them are known to be involved in early steps of coordinated gene expression. This may provide further support that there are broad interconnections between splicing factors and actively transcribed regions in the human genome. Similar investigation was performed for other RNA-binding proteins by Van Nostrand et al. [24]. To fully understand the roles of splicing factors we should also take into account genes that are translationally regulated by them. For SRSF1 it was shown that >1500 mRNAs are its translational targets, and many of these mRNAs are required for normal mitotic progression [55]. Moreover, in breast cancer, SRSF1 moves to the cytoplasm where it promotes the translation of *MYC* and other mRNAs [56]. These examples show how complex may be the roles of splicing factors in the control of gene expression at multiple levels, especially in cancer cells.

### Cell proliferation

Splicing factors are often overexpressed in many solid tumors compared to adjacent normal tissues. Thus, SRSF1 overexpression is observed in 13% of patients with breast cancer, 25% with colon cancer, and 25% with lung cancer [53]. An increased abundance of most splicing factors often correlates with increased tumor cell proliferation [57–60]. This effect can be implemented through the changes in alternative splicing of various mRNAs encoding oncogenes (PTEN [61], EGFR [62], BRAF [63]), activation of the transcription and translation of cell cycle genes [64] (Table 1). For example, hnRNP Q1 can bind to 5'-untranslated regions (UTRs) of *Aurora-A* mRNA thus regulating its translation, and subsequently increasing the proliferation of colorectal cancer cells [65]. Conversely, enhanced expression of the splicing factor FUS in hepatocellular carcinoma cells has been associated with inhibition of cancer progression. FUS binding to *LATS1/2* mRNA ensures its stabilization and activation of the Hippo signaling pathway, which regulates cell proliferation and apoptosis [66]. Our analysis of the ENCODE project data shows that depletion of most splicing factors leads to increased expression of CDKN1A, a cyclin-dependent kinase inhibitor 1, which is involved in TP53 mediated inhibition of cellular proliferation. Splicing of DHRS2, a dehydrogenase/reductase SDR family member 2, is often disrupted in response to splicing factor depletion. Normally, this protein attenuates MDM2-mediated TP53 degradation, leading to TP53 stabilization and accumulation of MDM2 and CDKN1A. These examples made it clear that many splicing factors can affect cell cycle regulation.

Considering that many splicing factors are also DNA or histone modifying enzymes, they can directly participate in chromatin remodeling, thus regulating the expression of oncogenes or tumor suppressor genes and, therefore, promote proliferation, migration, and invasion of cancer cells. This has been demonstrated for histone deacetylase SIRT1 [67, 68], DNA demethylase TET1 [69], histone deacetylase HDAC2 [70], histone methyltransferase SETD2 [71], RNA helicase DDX17 [72], and arginine methyltransferase CARM1 [73].

### Cell cycle progression

Changes in the expression of splicing factors disrupt the progression of the cell cycle and the process of division (Table 1) [34, 74, 75]. Most often, a change in the expression of spliceosome proteins results in cell cycle arrest at the G2/M phase. This effect can be accompanied by both an increase (LSm1, etc.) [76] and a decrease (CRNKL1, SNRPB, SRSF1) [55, 74] of expression of some splicing factors. It has been shown, that this effect may be dose-dependent on the expression level of the splicing factor. For example, depending on the degree of SNRPB knockdown, cell cycle arrest occurs at different stages: G2/M or an earlier stage of G1/S in case of more complete depletion. At a minimal level of knockdown, cells can even undergo mitosis in spite of a large number of mitotic defects [74].

Interestingly, expression of spliceosome genes and genes involved in the mitotic part of the cell cycle is simultaneously downregulated in cancer cells in response to stress factors, such as chemotherapy [77]. According to our analysis of the ENCODE project data, knockdown of more than one third of analyzed splicing proteins resulted in changes in the expression but not splicing of CDKN2B, JUNB, CCND2, BTG2, PIM1, etc., which are required for normal cell cycle regulation and cell proliferation (Supplemental Table 1).

Splicing factors may also be involved in the regulation of cell cycle progression and promote tumor cell survival in a splicing-independent manner. Thus, DDX5 can bind to mRNA of cyclin genes, regulating their nuclear export and stability. In addition, the interaction of DDX5 with a noncoding RNA *SUNO1* enhances binding of DDX5 to RNA polymerase II, which contributes to its stabilization on chromatin and transcription of cell cycle genes [78]. The hnRNP Q1 protein can upregulate the translation of the spindle assembly checkpoint genes and, in addition, induce the translation of the *Aurora-A* mRNA involved in the regulation of mitosis. Overexpression of hnRNP Q1 in tumor cells may contribute to tumorigenesis [79].

Numerous RNAi-based screens have revealed splicing factors that directly contribute to open mitosis. For example, the RBM10 protein regulates centriole duplication and its overexpression in tumor cells leads to cell cycle arrest in the M phase and the formation of a monopolar spindle due to disturbances in centriole duplication [80]. According to Pellacani and co-authors, splicing factors SF3A2 and PRP31 are necessary for normal chromosome segregation, as they regulate the interaction between kinetochores, spindle microtubules, and the essential kinetochore complex Ndc80 [81]. Similarly, depletion of splicing factors, such as NHP2L1/SNU13, SART1, MFAP1, CDC5L, SNW1, PRP19/PRPF19, or UBL5c, leads to defective chromosome segregation [82–85]. Interaction of U5 snRNP proteins (particularly EFTUD2 and SNRNP200) with cohesin is also important for mitotic progression [86].

Thus, splicing factors not only influence the progression of the cell cycle and cell division by regulating the expression and splicing of cell-cycle genes, but also by directly interacting with cohesin complex proteins, microtubules, and kinetochores. Marked changes in the expression of core spliceosome proteins invariably cause cell cycle arrest or may even lead to cell death.

### Migration and invasion

Changes in splicing factor levels entail changes in the rates of migration and invasion of tumor cells [87, 88]. These processes are often mediated by larger changes associated with the epithelial-mesenchymal transition (EMT). For example, overexpression of the RBM8A protein promotes migration and invasion of hepatocellular carcinoma cells by inducing the EMT via activation of the transcriptional regulator HDAC9 [89, 90]. Interestingly, U1 snRNA overexpression has the opposite effect on tumor cell migration and invasion [91]. Other examples are listed in Table 1. According to our analysis, depletion of many splicing factors (e.g., HNRNPK, PABPC1, PTBP1, RAVER1, RBM22, SF3B4, U2AF1, SRSF9) in the HepG2 cell line also leads to an increase in the expression of EMT inducers (TGF-β and CD44) as well as EMT-mediating transcription factors ZEB2, SNAI1, SNAI2, SOX18, and FOXC1 (Supplemental Table 1). In turn, depletion of HNRNPK, NONO, RAVER1, RBM22, and SFPQ leads to an increase in the expression of metalloproteinases MMP-9, MMP-12, and MMP-14, which are associated with invasion and metastasis in hepatocellular carcinoma [92]. Notably, depletion of these splicing factors also leads to an increase in the expression of a metalloproteinase inhibitor, TIMP1. However, there were no alterations in the splicing of the proteins listed above.

### DNA repair

While cellular responses to DNA damage are considered as critical determinants of cancer development, level of splicing factors in the cell are also crucial to this process (Table 1). A growing body of evidence shows that an increased abundance of splicing factors leads to higher levels of DNA repair proteins. RNF113A protein not only regulates alternative splicing of genes required for DNA damage response, but also stabilizes the levels of an antiapoptotic protein MCL-1 and prevents cell death in lung cancer cells treated with cisplatin [93]. Splicing factor CIRBP induces expression of HIF-1α via binding to the 3'-UTR of its mRNA to increase the mRNA stability in bladder cancer cells [94].

On the other hand, according to genome-wide siRNA screening data, knockdown of many splicing factors induces genomic instability due to uncoupling of transcription and splicing processes and formation of R-loops, which also leads to activation of replication stress and DNA damage response (DDR) in tumor cells. For example, this has been shown for splicing factors, such as NHP2L1/SNU13, MGC13125, SKIIP, and SF3A1 [95], ASF/SF2, hnRNP C1/C2, hnRNP K, SC35 [96], and SF3B1 [97]. In general, according to our analysis of the ENCODE project data, depletion of splicing factors leads to changes in the expression and splicing of most genes associated with the “DNA repair” GO term (Supplemental Table 1). Alterations in splicing of mRNAs encoding proteins associated with DNA damage response, in turn, are a source of gene mutations that reciprocally affect the functions of splicing proteins as well as the splicing process [98]. This effect can also be observed when the abundance of splicing factors is decreased in other ways, such as auto-repression or exporting them from the cell via extracellular vesicles [99].

A number of splicing factors can directly trigger the DNA damage response. For example, E3-ubiquitin ligase PRP19 is a sensor for single-strand breaks: it ubiquitinates RPA that provokes the ATRIP and ATR proteins recruitment to the damage sites [31]. FUS is recruited to double-strand break sites in a PARP-dependent manner and enhances DDR by interacting with histone deacetylase 1 (HDAC1) which is required for proper DNA repair [100]. It has also been shown that RNA polymerase II arrest at different transcription-blocking DNA lesions results in displacement of the core spliceosome resulting in initiation of ATM signaling [101].

Many splicing factors can interact with DNA repair proteins, stimulate their activity, and thus can direct cellular response to DNA damage. For example, it has been shown that the YB-1 protein can participate in almost all types of DNA repair due to interaction with such proteins as PCNA, MSH2, XRCC5 and DNA ligase IIIα, etc. [102]. It is also known that YB-1 has an increased affinity for DNA containing abasic sites or mismatches. By binding to such DNA regions, this protein promotes local melting of duplexes, which facilitates their repair [103]. Downregulation of a splicing factor E3 ubiquitin ligase RNF8 reduces ubiquitination of DNA damage sites in chromatin and suppresses subsequent recruitment of repair factors such as WRAP53β, RNF168, 53BP1, BRCA1, and RAD51 [104]. The interaction between hnRNP U and DNA glycosylase NEIL1 stimulates the activity of this enzyme, which is responsible for the recognition and removal of oxidized DNA bases [105]. RBMX/hnRNP G binds to DNA double-strand breaks, protects such regions from further degradation, and stimulates the non-homologous end joining system repair [106]. Similarly, a heterodimer of two splicing factors SFPQ–NONO stabilizes paired DNA ends and stimulates non-homologous end joining, forming a preligation complex together with the Ku protein [107]. RBMX/hnRNP G has also been shown to be a positive regulator of homologous recombination. It is shown to be accumulated at sites of DNA damage in a PARP1-dependent manner and promotes resistance to several DNA damaging agents [108].

### Cell death

Differentially expressed splicing factors exhibit various effects on programmed cell death regulation. Examples of apoptotic factors regulation through alternative splicing mechanisms are well discussed by Lin and co-authors [109]. Here, we have collected examples not related to changes in the splicing of anti-apoptotic and pro-autophagy genes (Table 1). According to our analysis of the ENCODE project data, only PRPF4 or MATR3 depletion led to changes in TP53 expression, while the depletion of other splicing factors affected the expression of known p53-response genes: IGFBP3, SERPINE1, CDKN1A, and THBS1 (Supplemental Table 1). A decrease in the abundance of DDX28, GPKOW, PUF60, RBM17, and RBM39 in cancer cells also downregulates expression of the EGR1 transcription factor, which regulates cell proliferation and cell death.

In response to 5-fluorouracil treatment, the DHX32 expression was downregulated and this was accompanied with chemoresistance acquisition of colorectal cancer cells. It may be in part due to the fact that DHX32 is less effectively involved in the processing of mitochondrial RNAs necessary for mitochondria-mediated apoptosis [110].

USP39 deubiquitinating enzyme (U4/U6.U5 snRNP component) regulates apoptosis through deubiquitination and stabilization of CHK2 [111] or activation of the AKT signaling pathway [112]. However, the effects of overexpression or knockdown of this protein vary in different cell lines. For example, in multiple lung cancer cell lines, downregulation of USP39 confers cancer cells resistance to chemo- and radiotherapy and, conversely, silencing of USP39 in the case of pancreatic cancer induces apoptosis and suppresses tumor growth [111, 112].

Overexpression of hnRNP K plays an important role in the radioresistance of colorectal carcinoma cells [113] where hnRNP K binds to phosphorylated p53 in the cytoplasm. This interaction contributes to the stabilization of various mRNAs resulting in radioprotective effect. In particular, binding of hnRNP K to the CU-rich region in thymidine phosphorylase *TYMP* mRNA results in prolonging the half-life of mRNA molecules and thereby in increasing protein levels of thymidine phosphorylase. As shown for nasopharyngeal carcinoma cells, such thymidine phosphorylase induction allows cells to resist hypoxia-induced apoptosis [114]. Similarly, SF2/ASF stabilizes mRNA of the anti-apoptotic protein survivin, thus increasing its translation [43].

Downregulation of SIRT1 blocks acetylation of the transcription factor FoxO1, reduces the number of autolysosomes in the cell, and thereby inhibits autophagy in pancreatic cancer cells [115]. Inhibition of SIRT1/2 also promotes the survival of lung cancer cells by triggering autophagy and blocking apoptosis via acetylation of HSPA5 and subsequent activation of ATF4 and DDIT4 to inhibit the mTOR signaling pathway [116]. On the contrary, DDX5 overexpression promotes autophagy and reduces cancer cell growth and tumorigenesis in HepG2 and Huh7 cells. DDX5 binds to autophagic receptor p62, promoting its activation. It decreases p62/TRAF6-mediated lysine 63-linked ubiquitination of mammalian target of rapamycin (mTOR) and subsequently inhibits the mTOR signaling pathway [117]. Similarly, exogenous overexpression of the splicing factor TDP-43 activates autophagy and suppresses stress-induced apoptosis via enhancing the expression of histone deacetylase 6 (HDAC6) [118]. The effect of changes in the expression of splicing factors on autophagy also largely depends on the stage of cancer. Thus, at the early stages of tumorigenesis, autophagy can suppress tumor development, while at later stages it can promote the survival of tumor cells and protect them from various therapeutic interventions [119].

### Cellular senescence

Cellular senescence is one of the most important biological processes, which activation protects cells from malignant transformation [120]. Downregulation of many splicing factors is known to contribute to cellular senescence. The transition of a cell to a state of pseudo-senescence is often induced by the formation of p53β, an alternatively spliced isoform of p53 [121]. Other examples of the role of alternative splicing in the induction of cellular senescence are well described in several reviews [122–126]. Recent studies have shown that splicing factors can also trigger cellular senescence through functions unrelated to alternative splicing (Table 1). In particular, it is known that SRSF3 can contribute to cellular senescence by providing alternative polyadenylation of transcripts at proximal poly(A) sites. After SRSF3 knockdown, mRNAs with shorter 3'-UTRs are accumulated in the cell which stimulates the production of more proteins, possibly by escaping the miRNA targeting. Functional annotation of genes whose mRNAs are subject to such alternative polyadenylation showed the predominance of senescence-associated pathways, which was also reflected in the cell phenotype [127]. Depletion of another splicing factor, DDX24 protein (ATP-dependent RNA helicase), impairs its interaction with p300. This leads to increased p300-dependent p53 acetylation, induction of cell cycle arrest, and cellular senescence [128].

## Subcellular relocation of spliceosome components affect cellular processes associated with cancer progression

It is imperative for the cells not only to have the appropriate expression levels of splicing factors but also their proper localization for normal functioning. Perturbations in the subcellular localization of antagonist splicing factors (ASF/SF2 and hnRNP A1, etc.) lead to a change in their ratio in the nucleus, which affects the regulation of alternative splicing of various proteins [15, 129]. Ectopic localization of spliceosome components may be associated with cancer since, depending on the subcellular localization, splicing factors can function either as oncogenes or as tumor suppressors (Table 1).

Different subcellular populations of SIRT1 may have opposite roles in modulating cell apoptosis [115]. Thus, it has been shown that cytoplasmic (i.e., ectopic) SIRT1 localization is associated with a shift in the phenotype of ovarian carcinoma cell line IGROV1 from mesenchymal to more epithelial type, accompanied by inhibition of migration and invasion processes. Different SIRT1 subcellular localization affected the acetylation levels of three EMT-related proteins (CK-18, vimentin and desmoplakin) [130].

Another splicing factor that may have different functions depending on its subcellular localization is PTBP1 (hnRNP I). In the nucleus, PTBP1 regulates the splicing of many transcripts whose AS changes correlate with malignant transformation in colon cancer [131], pancreatic cancer [132], and ovarian cancer [133]. Functioning in the cytoplasm, PTBP1, on the contrary, can inhibit the proliferation and invasion of tumor cells by binding to mRNAs of various tumor suppressors, which leads to the stabilization of such transcripts (tumor necrosis factor CD154 mRNA in activated T lymphocytes [134]); activation of IRES-mediated translation (cyclin-dependent kinase inhibitor protein p27 [135], apoptotic protease activating factor 1, Apaf-1 [136]). In addition, binding of PTBP1 with mRNAs of oncogene AXL leads to their degradation [137].

In recent years, experimental techniques aimed to decipher the localization of proteins at various scales and resolutions, including a high resolution mass spectrometry-based approaches such as spatial proteomics and proximity labeling, being actively developed [138, 139]. With help of such advanced technologies, researchers will be able to get a comprehensive picture of splicing factors subcellular localization and its relationship to protein function in the near future.

## Spliceosome components participate in intercellular communication

Spliceosome components were previously found to be secreted by dying cancer cells in response to cellular stress induced by chemotherapeutic drugs or hypoxia [6, 7, 77, 140]. However, the mechanisms of this secretion still remain unclear. According to the currently available data, it can be presumed that the reason for the secretion of these nuclear proteins may be due to the change in their subcellular localization during the formation of stress granules under different stresses. hnRNP A1, as well as a number of other hnRNPs and SR proteins, can be relocated from the nucleus to the cytoplasm and accumulated in stress granules in response to ultraviolet-C irradiation, heat shock, osmotic shock, hypoxia, and oxidative stress [141–143]. In addition to stress, the overexpression of some splicing factors (SRSF1, SRSF3) also leads to their accumulation in stress granules [143]. In addition, snRNAs have been shown to relocate to the cytoplasm in compromised conditions induced by sodium arsenite, thapsigargin, cisplatin, or irradiation [7, 144]. It is important that significant genome-wide splicing abnormalities are observed as a result of the incorporation of immature snRNAs into the spliceosome which lack the processing stage in the cytoplasm [145].

Spliceosome components can be secreted by dying tumor cells as part of extracellular vesicles under the effect of various stress factors (γ-irradiation, drugs, heat) [8, 146]. Once internalized in neighboring tumor cells, splicing factors can significantly contribute to the molecular events occurring in the recipient cell and also confer their resistance to chemotherapeutic agents [6, 7, 45, 147]. Thus, after internalization, exogenous RBM11 is transported to the nuclei of recipient glioblastoma cells and changes the splicing of MDM4 and Cyclin D1 towards the expression of pro-oncogenic isoforms [7]. Under oxidative stress, tumor cells can secrete splicing factor YB-1 into the extracellular space. Interestingly, this has an antiproliferative effect on receiving Caco-2 tumor cells through increase in the p21WAF protein level, decrease in the ∆Np63α protein level, and arrest of the cell cycle at the G2/M phase [45]. Depending on the method of internalization, the fate of the contents of extracellular vesicles may be different. In any case, exogenous proteins and RNA entering the cell destabilize the processes inside recipient cells, which requires further exploration.

## Autoregulation of splicing factors

**Fig. 3:**
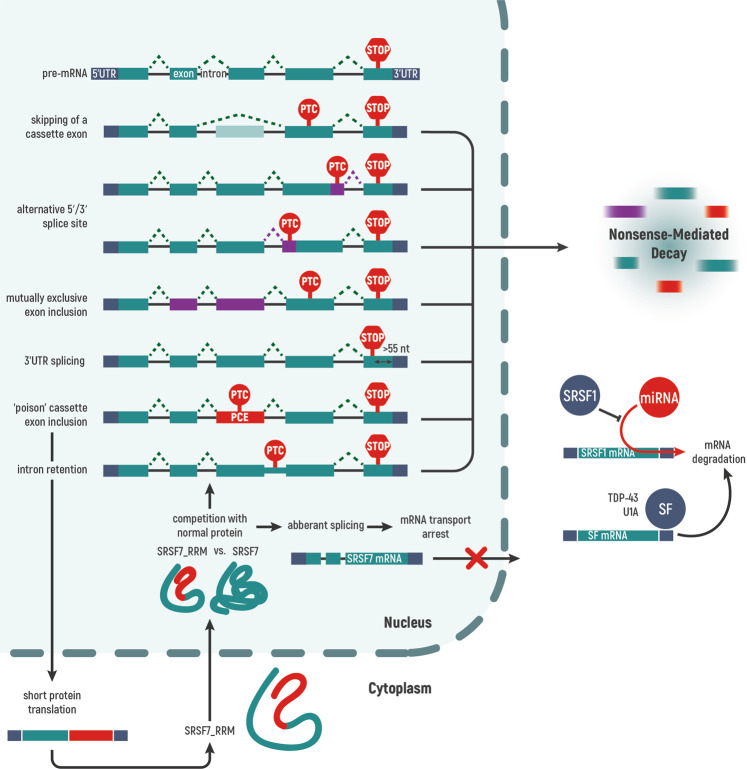
Known mechanisms of autoregulation of splicing factors. Splicing factors negatively autoregulate their own synthesis by promoting unproductive splicing of their own transcripts. Alternative splicing may create a full-length productive isoform that encodes a functional protein or may result in a premature termination codon (PTC). Transcripts with PTC are committed to nonsense-mediated mRNA-decay (NMD). Following events can lead to PTC: frameshift due to exon skipping; usage of an alternative 5' or 3' splice site with an in-frame PTC; frameshift due to inclusion of mutually exclusive exons (or none of them); splicing in the 3' untranslated region (UTR), at a position located >55 nucleotides downstream of the stop codon (STOP), creating a premature context that triggers NMD; retention of PTC-containing exon (also known as poison cassette exon, PCE); or retention of the intron with an in-frame PTC. Transcripts that include poison cassette exon can be NMD-resistant. In the case of SRSF7, negative autoregulation occurs due to the formation of functionally defective truncated protein or by the production of RNA isoforms, which are sequestered in the nucleus. The U1A protein is involved in autoregulation, preventing productive 3' end processing and polyadenylation. In the case of the TDP-43 splicing protein, it binds to GU-rich sequences in the 3'-UTR of its own mRNA, which causes its degradation. As an example of a positive feedback loop, the SRSF1 protein can compete with *Mir505-3p* for binding to its own mRNA, thereby inhibiting its own degradation. Introns are represented as black lines and exons as green boxes.

Since splicing factors perform key functions in the cell, their expression is regulated through a variety of mechanisms and at different levels. Temporary or permanent overexpression of splicing factors may destabilize different processes in tumor cells. In various cell line models, it was unexpectedly observed that exogenous overexpression of splicing factors leads to inhibition of their endogenous expression. However, the positive feedback regulation was also shown. A temporary increase in the abundance of spliceosome components in the cell may occur due to their penetration the recipient cell as part of extracellular vesicles from the tumor microenvironment. For example, it has been shown that exogenous RBM11 can increase the expression of endogenous RBM11 by binding to its mRNA [7]. In connection with this new data, in this review we summarize the currently known mechanisms of splicing factors autoregulation (Fig. 3). Other mechanisms, such as regulation of expression by transcription factors, miRNAs and long non-coding RNAs, as well as regulation by post-translational protein modifications, are well described in a recent review by Du and co-authors [54].

Most of the autoregulation mechanisms of splicing genes are reduced to feedback loops. This is due to the fact that RNA-binding proteins (RBPs) bind to and directly affect their own mRNAs, controlling their expression [148–150]. The question of the existence of positive feedback loops (feedforward loops) at the posttranscriptional level remains open. An example of a positive feedback loop, in addition to RBM11, has been shown for the SRSF1 protein [151]. In tumor cells, SRSF1 can compete with *Mir505-3p* for binding to its own mRNA, thereby inhibiting its own degradation.

A typical process of autoregulation of splicing factors and other RNA-binding proteins is Alternative Splicing coupled with Nonsense-mediated mRNA Decay (AS-NMD). This mechanism is also known as Regulated Unproductive Splicing and Translation (RUST). During this process, alternative splicing leads to mRNA degradation due to the formation of premature stop codon (PTC). AS-NMD is an additional gene expression control at the post-transcriptional level that can compensate for the increase in protein abundance in the cell due to the formation of NMD-targeted isoforms [152]. In the human body, more than one third of alternative splicing events are believed to result in PTC, which is a trigger for mRNA degradation by NMD [153, 154]. A codon is a PTC if it is >50 nt upstream of the final exon–exon junction as a result of alternative splicing [152]. The reason for this phenomenon (Fig. 3) may be (1) the inclusion of ‘poison’ cassette exon (PCE), (2) intron retention, (3) alternative 5'/3' splice site, (4) skipping of a cassette exon, (5) inclusion of both exons that are normally mutually exclusive, or (6) the normal stop codon becoming a PTC if the introns are >55 nt apart in the 3'-UTR region [153]. It is noteworthy that a large number of AS-NMD events are associated with ultraconserved genomic elements and share similarities between different types of living organisms and different groups of splicing factors, which underscores the importance of this process.

Bioinformatics analysis has shown that transcripts of RBPs, and particularly of splicing-related RBPs, tend to undergo NMD more frequently than transcripts of other protein-coding genes [155]. Moreover, according to crosslinking-immunoprecipitation (CLIP)-seq data, RBPs that have been shown to have at least one NMD-exposed transcript tend to bind to their own mRNA more frequently, than RBPs without NMD-exposed transcripts [155]. Thus, splicing-related RBPs can statistically more often regulate their own expression through AS-NMD. The possibility of premature termination codon formation in their mRNA molecules, which further triggers NMD, has been shown for most of the SR genes [154]. Table 2 summarizes these and other examples of autoregulation of splicing factor gene expression by AS-NMD.

**Table 2:** Known mechanisms of autoregulation of splicing factors.

Protein Family	Protein	Mechanisms of autoregulation	References
**SR proteins**	SRSF1 (ASF/SF2)	alternative splicing associated with NMD or nuclear retention; protein overexpression reduces the translational efficiency of its own mRNA	[150, 180]
	SRSF2 (SC35)	alternative splicing associated with NMD	[181]
	SRSF3 (SRp20)	alternative splicing associated with NMD; alternative splicing resulted in protein isoform with impaired function	[182]
	SRSF4 (SRp75)	alternative splicing associated with NMD (?)	[183]
	SRSF5 (SRp40)	alternative splicing associated with NMD	[184, 185]
	SRSF7 (9G8)	alternative splicing associated with NMD or nuclear retention	[156]
	SRSF10	alternative splicing resulted in protein isoform with impaired function	[163]
	TRA2B	alternative splicing associated with NMD	[186]
**hnRNPs**	hnRNP A2B1	alternative splicing associated with NMD	[187]
	hnRNP I (PTBP1)	alternative splicing associated with NMD	[188, 189]
	hnRNP L	alternative splicing associated with NMD	[190]
	FUS/TLS	alternative splicing resulted in protein isoforms with different cellular localization	[191]
	hnRNP M	alternative splicing associated with NMD (bioinformatic prediction)	[155]
	TDP43	alternative splicing associated with NMD or nuclear retention and exosome-mediated decay	[161, 192]
**Other**	Fox proteins (Fox-1, −2, −3)	alternative splicing resulted in protein isoforms which antagonize Fox activity	[157]
	MBNL1	alternative splicing associated with NMD; alternative splicing resulted in protein isoforms with different cellular localization	[158, 193]
	RBM10	alternative splicing associated with NMD	[194]
	RBM39	alternative splicing associated with NMD (bioinformatic prediction)	[155]
	SFPQ	alternative splicing associated with NMD	[155]
	TIA1	alternative splicing associated with NMD	[195]
	U1A	binding to and inhibiting the polyadenylation of its own pre-mRNA (through PAP inhibition)	[160]
	U2AF1	alternative splicing associated with NMD	[155]
	U2AF2	alternative splicing associated with NMD (bioinformatic prediction)	[155]

Transcripts that include PCE can be NMD-resistant. In such a case, negative autoregulation of splicing factors can also occur due to the formation of non-functional or functionally defective truncated protein [152]. For example, in the case of overexpression, full-length SRSF7 presumably binds to SRSF7 transcripts with included PCE, which stimulates translation from Split-ORF2 and downstream of the PTC, followed by the formation of truncated protein SRSF7_RRM without RS domain. By accumulating in the cell, truncated SRSF7 competes with the full-length protein for binding to the 5' splice site, which leads to the retention of introns 3 and 5 in the *SRSF7* mRNA molecule [156]. In another example, Fox-induced splicing produces RNA binding protein fox-1 homolog 1 and homolog 2 that lack a functional RNA binding domain (RRM). Such proteins bind weakly to RNA and act as repressors of Fox-dependent splicing [157]. A high concentration of MBNL1 stimulates its interaction with its own mRNA and excludes exon 1. This leads to a decrease in the efficiency of further translation due to the complication of coordination with polysomes. As a result, a truncated, unstable, and less active protein with two zinc fingers can be formed instead of the four zinc fingers required for mRNA recognition [158, 159].

In addition to regulation through the AS-NMD mechanism, some splicing factors produce RNA isoforms during autoregulation, which are then sequestered in the nucleus. Thus, above-mentioned SRSF7 transcripts with retained introns 3 and 5 are retained in the nucleus. During SRSF7 overexpression, SRSF7 transcripts, both containing introns and fully spliced and polyadenylated, retain in the nucleus and form nuclear bodies [156].

Another mechanism of autoregulation is realized through the binding of the splicing factor to the 3'-UTR sequence of their own transcript, which leads to mRNA destabilization. The U1A protein is involved in autoregulation, preventing productive 3'-end processing and polyadenylation [160]. Two molecules of the U1A protein bind to a certain element in the 3'-UTR of their own mRNA located at a conservative distance from the polyadenylation site, and at the same time contain a polyadenylation inhibition element. Regulation involves inhibition of poly(A) tails formation due to decreasing poly(A) polymerase (PAP) enzymatic activity. In the case of the TDP-43 splicing protein, it binds to GU-rich sequences in the 3'-UTR of its own mRNA, which causes its degradation most likely by RNA exosome [161, 162].

Another interesting study shows the connection between minor and major spliceosomes where minor spliceosome activity directly controls SRSF10 levels, which results in altered expression levels of other SR proteins. When there is a dominant activity of the major spliceosome, an unproductive SRSF10 variant with included exon 3 accumulates in the cell. If the activity of the minor spliceosome is high, the resulting splice isoform contains exon 4 and is protein-coding [163].

These examples of autoregulatory circuits (summarized in Fig. 3) demonstrate that autogenous regulation of various splicing factors is rather simple and robust. It remains to be determined how broadly such regulation is employed by other splicing factors and how we can harness the knowledge for therapeutic manipulations.

## Conclusions

RNA splicing is being intensively studied, but there are still many challenges in this field in regard to kinetics and mechanisms of this process in vivo, either under normal conditions, or especially in response to stress. The splicing machinery includes hundreds of proteins and several snRNAs whose expression needs to be precisely controlled for normal cell physiology. By introducing significant perturbations in the splicing process, cancer cells generate many transcript isoforms, some of which can be advantageous for their survival. To overcome various stresses, cancer cells may exploit not only splicing activity of spliceosome components but also their splicing-independent functions. By virtue of their nature, many spliceosome proteins are able to bind to different classes of proteins and nucleic acids. This enables regulation of almost any process in the cell in a simple and energy-efficient way, in particular, autoregulation of the splicing process itself, adjusting it to the current needs of the cell (e.g., cancer progression, chemoresistance acquisition). The role of the splicing machinery is widening and is no longer limited to its known functions.

Spliceosome components and individual splicing products have been considered as potential targets for anticancer therapy since the early 2000s [164]. Currently known strategies for the control of the alternative splicing include: removal of unwanted transcripts (various RNA editing approaches [165]); specific inhibition of splicing factors, for example, RBM39 [166], SRSF6 [167], PRMT5 [168]; using various spliceosome inhibitors for global modulation of RNA splicing (H3B-8800 [169] and Indisulam [170] are undergoing clinical trials). The exact mechanisms of cytotoxic action of splicing inhibitors on cellular processes are still poorly understood, but the main consequences of spliceosome blockade can be noted: unproductive splicing and subsequent nonsense-mediated decay of DNA repair transcripts (CHEK2) [171] or the generation of pro-apoptotic protein isoforms (Mcl-1S) [172]; a large number of transcripts with retained introns forming an excess of double-stranded RNA in cytoplasm with the following activation of antiviral signaling and apoptosis of cancer cells [173]. Based on it, a number of new therapeutic combinations of splicing inhibitors with CHEK2 inhibitors or ADAR enzymes can be suggested. The use of splicing modulators for drug-induced neoantigen production and enhancing tumor immune recognition is also actively studied [174]. Moreover, it has been proposed synthetic constructs that were differentially spliced in cells with cancer-associated mutations in splicing factors to allow for cancer cell-specific toxic protein production [175]. As shown in this review, splicing-independent functions of spliceosome components also need to be taken into account in order to counteract their oncogenic activity. Promising approaches could include targeting of SR proteins to holistically modulate their roles in transcriptional, co-transcriptional, and post-transcriptional regulation pathways [176, 177]; or specific targeting of splicing factors, such as SRSF1, hnRNP A1 and hnRNP D, which regulate IRES-directed mRNA translation of different proto-oncogenes [56, 178]. In the context of the growing body of evidence demonstrating different splicing-independent functions of spliceosome proteins, we assume that new therapeutic approaches will arise based on a combination of splicing modulators and traditional immuno- and chemotherapeutic drugs [77, 179].

## Supplementary information

Supplemental Methods

Supplemental Table 1

Supplemental Table 2

Supplemental Table 3

Supplemental Table 4

Reproducibility Checklist

## Data Availability

Datasets described here can be obtained from the ENCODE project website at http://www.encodeproject.org via accession numbers in Supplemental Table 4.
