# Supplementary material for: Non-canonical functions of spliceosome components in cancer progression
Source: Cell Death Dis. 2023 Feb 2;14(2):77. doi: 10.1038/s41419-022-05470-9 (PMC9895063; doi:10.1038/s41419-022-05470-9)
Supplement: Supplementary file 1 — Supplemental Methods [file 41419_2022_5470_MOESM1_ESM.pdf]

Bioinformatics analysis of data from publicly available database

1. Published unprocessed RNA-seq reads of HepG2 cells treated with an shRNA knockdown against 76 different splicing factors and control HepG2 cells were downloaded from the ENCODE portal (https://www.encodeproject.org/). Quality filtering of the sequence reads was performed using Trimmomatic software (v. 0.38). Transcript-level abundances were quantified for each sample using a quasi-mapping approach with Salmon (v. 0.12.0) on the Gencode reference transcriptome GRCh37.p13 with default parameters. Then transcript-level abundances were aggregated to the gene-level abundances using Tximport R package (v. 1.16.1). Differential expression analysis was performed with DESeq2 (v 1.28.1). P-values were adjusted by the Benjamini-Hochberg method to control False Discovery Rate (FDR) at 0.05. Significant differentially expressed genes were defined as having a FDR threshold < 0.05 and absolute log-fold change threshold>=1.

2. rMATS JunctionCountsOnly files of HepG2 cells treated with an shRNA knockdown against 75 different splicing factors were downloaded from the ENCODE portal (https://www.encodeproject.org/). Significant alternatively spliced (AS) events were defined as having a FDR threshold < 0.05 and IncLevelDifference value > 5%.

3. For eCLIP and ChIP-seq data we downloaded bed narrowPeak files of HepG2 cells treated with an shRNA knockdown against 51 different splicing factors from the ENCODE portal (https://www.encodeproject.org/). Peak annotation was performed with ChIPseeker (v 1.32.1). Only Chip-seq peaks containing the promoter in the 1 kb surrounding regions of TSS were considered.

4. AS events, differential expression changes and were considered for the sets of genes included in the following Gene Ontology Terms: cell population proliferation (GO:0008283; 2009 genes in total), cell migration (GO:0016477; 1525 genes in total), cell cycle (GO:0007049; 1727 genes in

total), DNA repair (GO:0006281; 518 genes in total), cell death (GO:0008219; 2109 genes in total), cellular senescence (GO:0090398; 93 genes in total).

5. To identify significant enrichment for each splicing factor between RNA - binding activities and expression changes upon knockdown, genes with significantly enriched eCLIP signal were overlapped with genes with significantly altered expression upon knockdown. To explore significant enrichment for each splicing factor between DNA - binding activities and expression changes upon its knockdown, genes with significantly enriched ChIP-seq signal in promoter regions were overlapped with genes with significantly altered expression upon knockdown. To test the possibility that AS splicing changes might be associated with significantly altered expression upon knockdown against splicing factors, genes with significant changes in the splicing pattern were overlapped with genes with significantly altered expression upon knockdown. Significance was determined by two-sided Fisher's exact test (p-value < 0.05 and the odd ratio confidence interval should not include 1).
